# Outpatient Treatment of Postbariatric Hypoglycemia With Canagliflozin

**DOI:** 10.1016/j.aed.2025.03.008

**Published:** 2025-04-10

**Authors:** Eugene Juin Yih Looi, Helen Margaret Lawler

**Affiliations:** Department of Endocrinology, Metabolism and Diabetes, University of Colorado School of Medicine - Anschutz Medical Campus, Aurora, Colorado

**Keywords:** post-bariatric hypoglycemia, canagliflozin, SGLT-1/2 inhibitor

## Abstract

**Background/Objective:**

The goal of this study was to examine canagliflozin’s efficacy in treating postbariatric hypoglycemia (PBH) in a real-world setting. We hypothesized that canagliflozin, a sodium-glucose cotransporter 1/2 inhibitor, would reduce postprandial hyperglycemia and hypoglycemia.

**Case Report:**

Retrospective analysis was performed on continuous glucose monitor data of 4 patients (3 females and 1 male) with PBH from Roux-en-Y gastric bypass who took canagliflozin 300 mg daily. The number of postprandial hyperglycemic (180-250 mg/dL and >250 mg/dL) and hypoglycemic (54-69 mg/dL and <54 mg/dL) episodes in a 4-week period before canagliflozin was started and a 4-week period on canagliflozin was compared using paired t-test statistics. Patients’ subjective reports of PBH symptoms prior to and on canagliflozin were obtained from review of chart notes.

**Discussion:**

Although canagliflozin was well tolerated with 1 reported urinary tract infection, canagliflozin did not significantly reduce hypoglycemic episodes or attenuate postprandial hyperglycemic excursions depicted on continuous glucose monitor. Subjectively, most patients did not report improvement in PBH symptoms. This led to discontinuation of canagliflozin in 3 of the 4 cases.

**Conclusion:**

Canagliflozin was not found to reduce postprandial hyperglycemia or hypoglycemia in PBH. Because sodium-glucose cotransporter 1 inhibition is brief and transient, it is likely that the postulated beneficial effects of canagliflozin may depend on timing of medication administration.

**Highlights**

• Canagliflozin use in a real-world environment did not significantly reduce postprandial hyperglycemia or hypoglycemia in patients with postbariatric hypoglycemia (PBH)
• Canagliflozin’s sodium-glucose cotransporter 1 inhibitory effect is brief and transient and likely the reason that it was ineffective in treating PBH in a real-world setting
• Further research is needed to examine canagliflozin as a potential treatment for PBH; yet, based on our results, we do not recommend its off-label use

**Clinical Relevance**

There are limited treatment options for postbariatric hypoglycemia (PBH). Canagliflozin, a sodium-glucose cotransporter 1/2 inhibitor, has been found to reduce postprandial hyperglycemia and hypoglycemia in recent studies. This case series explores the real-world, clinical use of canagliflozin in treating PBH.

## Introduction

Postbariatric hypoglycemia (PBH) is a complication that develops after bariatric surgery and has been attributed to inappropriate and exaggerated insulin secretion and a blunted glucagon response to hypoglycemia.^1^, ^2^, ^3^ Bariatric surgeries such as vertical sleeve gastrectomy and Roux-en-Y gastric bypass (RYGB) result in altered gastrointestinal anatomy, which causes rapid nutrient transit and absorption in the small intestine, leading to early appearance of high glucose levels, stimulating glucagon-like peptide-1 (GLP-1) and insulin secretion.^3^ After RYGB, there is also increased expression of intestinal glucose transporters, which may contribute to the development of postprandial hyperglycemia, which stimulates insulin release.^4^

The goal of PBH treatment is to reduce the frequency and severity of postprandial hypoglycemia.^3^ First-line treatment involves dietary modification that restricts simple carbohydrates and increases protein intake, aiming to diminish the consequent rise in insulin in response to the rapid digestion and absorption of simple carbohydrates.^1^ A small case report also showed improvement in PBH with uncooked corn starch.^5^ Medical therapeutic options remain scarce and are limited to off-label or experimental use with varying degrees of success depending on clinical response, adherence, and side effects.^3^ These medications include acarbose, GLP-1 antagonists, somatostatin receptor agonists, and diazoxide.

The small intestine has sodium-glucose cotransporter (SGLT-)1 transporters, which play an important role in postprandial glucose absorption.^6^ Canagliflozin, an SGLT-2 inhibitor, has been found to also inhibit SGLT-1 transporters at doses of >200 mg.^7^ Canagliflozin 600 mg administered 10 minutes before a liquid mixed meal tolerance test delayed glucose absorption and suppressed early GLP-1 and glucose-dependent insulinotropic polypeptide responses, leading to decreased insulin release.^8^ In another trial, canagliflozin 300 mg daily for 2 weeks reduced hypoglycemic events by almost 86% after an oral glucose tolerance test.^9^ These studies demonstrate that SGLT-1/2 inhibitors represent a possible therapeutic option in patients with PBH.

To date, there are no known real-world outcomes reported regarding the efficacy of canagliflozin in ameliorating PBH. As such, the goal of this case series was to retrospectively examine the use of canagliflozin in patients with PBH and determine whether canagliflozin decreased postprandial glycemic excursions and/or decreased postprandial hypoglycemic episodes. Overall, this case series aimed to contribute to the existing body of knowledge regarding therapeutic options for PBH.

## Case Report

### Clinical Characteristics

Four patients (3 females and 1 male) were included in this case series, with the mean age of 45.8 years (Table 1). All had undergone RYGB for class II or III obesity (mean body mass index, 45.3 kg/m^2^), and only 1 patient (case 2) had type 2 diabetes mellitus prior to surgery, which went into remission after surgery. PBH was diagnosed after a mean duration of 2.3 years after surgery. Most had tried prior medications for PBH except for case 1. The mean duration of canagliflozin use was 2.3 months.

**Table 1:** Clinical Characteristics of Patients With Postbariatric Hypoglycemia

Clinical characteristics	Case 1	Case 2	Case 3	Case 4
Age (y)	35	48	53	47
Sex	Female	Female	Male	Female
Surgery	RYGB	RYGB	VSG converted to RYGB	RYGB
Maximum weight	282 lbs BMI, 41.6 kg/m^2^	300 lbs BMI, 51.5 kg/m^2^	250 lbs BMI, 35 kg/m^2^	310 lbs BMI, 53.2 kg/m^2^
Nadir weight	166 lbs BMI, 24.5 kg/m^2^	124 lbs BMI, 21.3 kg/m^2^	114 lbs BMI, 17.6 kg/m^2^	199 lbs BMI, 34.2 kg/m^2^
PBH onset after surgery	2 y	3 y	3 y	1 y
DM prior to surgery/now	No/no	Yes/no	No/no	No/no
Prior PBH medication	None	Acarbose Diazoxide Dulaglutide	Acarbose	Acarbose Empagliflozin

Abbreviations: BMI = body mass index; DM = diabetes mellitus; PBH = postbariatric hypoglycemia; RYGB = Roux-en-Y gastric bypass; VSG = vertical sleeve gastrectomy.

### Methods

A 4-week period of continuous glucose monitor (CGM) data before and another 4-week period of CGM data after canagliflozin was started were obtained from CGM brand-specific cloud-based interface. Postprandial was defined as a rise in the blood glucose (BG) level of >20 mg/dL from baseline BG level, and the postprandial period was defined as the 4-hour period after the start of the BG rise.^10^ The following events were manually counted for each patient: number of (1) level 1 postprandial hypoglycemic events (defined as a BG level of 54-69 mg/dL),^11^ (2) level 2 postprandial hypoglycemic events (defined as a BG level of <54 mg/dL),^11^ (3) postprandial hyperglycemic events with a BG level of 180 to 250 mg/dL, and (4) postprandial hyperglycemic events with a BG level of >250 mg/dL. Chart review revealed that all patients were educated on possible side effects and were instructed to eat mixed meals high in protein with a goal of 30 g of carbohydrates/meal (not less) to reduce the risk of euglycemic diabetic ketoacidosis while on canagliflozin. Paired t-test statistical analysis was performed on the data collected.

### Efficacy of Canagliflozin

Collectively, CGM analysis showed no significant reduction in the number of postprandial hyperglycemic episodes with a BG level of 180 to 250 mg/dL (*P* = .48), postprandial hyperglycemic episodes with a BG level of >250 mg/dL (*P* = .41), level 1 postprandial hypoglycemic events (*P* = .27), and level 2 postprandial hypoglycemic events (*P* = .14).

**Fig. 1:**
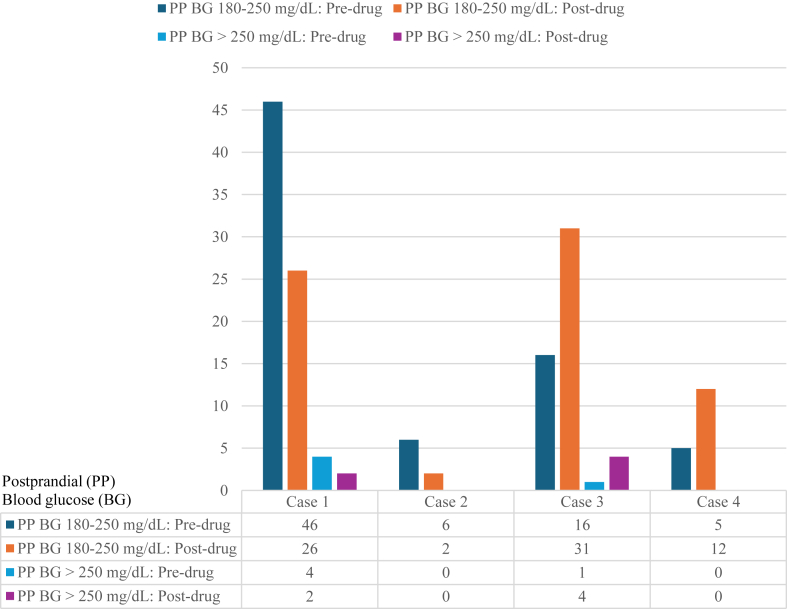
Number of postprandial hyperglycemic episodes before and after canagliflozin 300 mg daily.

All patients, except case 1, reported no reduction in postprandial hyperglycemic episodes (Table 2). The patient in case 1 observed decreased hyperglycemic episodes, which correlated with CGM data showing a 44% reduction (Fig. 1). The patients in cases 3 and 4 noted an overall increase in the number of hyperglycemic episodes, which was consistent with CGM data.

**Table 2:** Clinical Response to Canagliflozin 300 mg Daily

Canagliflozin	Case 1	Case 2	Case 3	Case 4
Months on canagliflozin	2 mo	2 mo	2 mo	3 mo
Side effects on canagliflozin	None	None	None	UTI
Subjective report of BG on canagliflozin	Reduction in postprandial BG excursions, no improvement in postprandial hypoglycemic episodes	No reduction in postprandial BG excursions, slight reduction in the number and severity of postprandial hypoglycemic episodes	Overall increase in postprandial glycemic excursions and hypoglycemic episodes	Overall increase in postprandial glycemic excursions and hypoglycemic episodes

Abbreviations: BG = blood glucose; UTI = urinary tract infection.

**Fig. 2:**
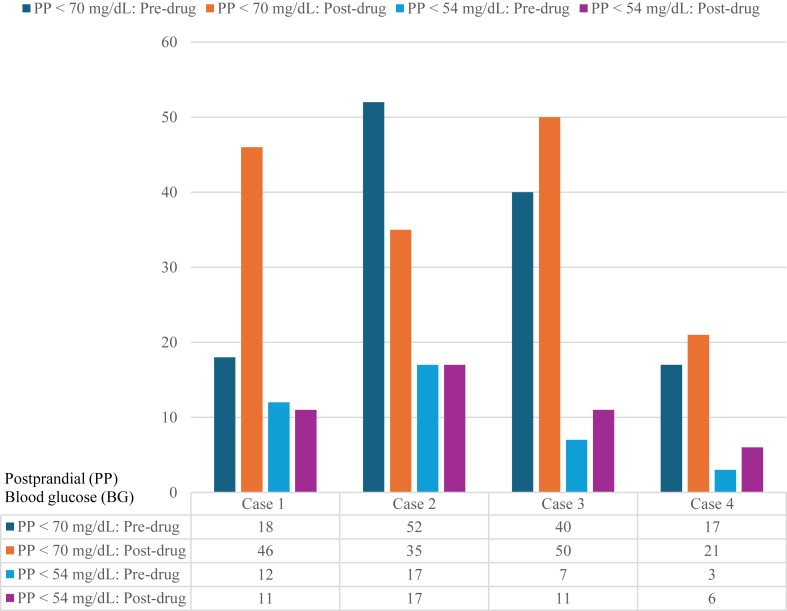
Number of postprandial level 1 and level 2 hypoglycemic episodes before and after canagliflozin 300 mg daily.

All patients, except case 2, reported no reduction in postprandial level 1 and level 2 hypoglycemic episodes (Table 2). In fact, the patients in cases 3 and 4 reported an overall increase in hypoglycemic episodes, consistent with CGM data (Fig. 2). The patient in case 2 noted a slight reduction in hypoglycemic episodes, which was supported by CGM data showing a 33% decreased frequency of level 1 hypoglycemia but no change in level 2 hypoglycemia (Fig. 2).

Canagliflozin was well tolerated other than 1 episode of urinary tract infection in case 4 that did not result in medication discontinuation. No other side effects were reported. Canagliflozin was discontinued in cases 2 to 4 because patients felt that they did not experience clinical benefit.

## Discussion

In this retrospective case series, canagliflozin 300 mg daily was not found to ameliorate PBH. No significant difference was observed in the number of postprandial hyperglycemic and hypoglycemic episodes depicted on CGM. It is likely that the SGLT-1 inhibitory effect of canagliflozin was not reproduced in a real-world setting because SGLT-1 inhibition may be brief and transient, occurring within the first few hours of canagliflozin administration. This observation is supported by the finding that canagliflozin’s inhibition of SGLT-1 predominantly occurs in the first 2 hours after caloric consumption in healthy subjects and that postprandial reductions in glucose and insulin excursions were only observed after the meal consumed closest to canagliflozin administration and not in subsequent meals.^12^^,^^13^ In the 2 trials demonstrating benefit, canagliflozin was administered 10 and 60 minutes before caloric intake.^8^^,^^9^ Rodent studies of the pharmacokinetic profile of oral mizagliflozin, a selective SGLT-1 inhibitor, also support evidence that SGLT-1 inhibition has a rapid onset, which then quickly dissipates. Mizagliflozin’s half-life was 1.14 hours, and the bioavailability was only 0.02%, indicating poor absorption and rapid clearance from circulation.^14^ Due to the case series’ retrospective nature, timing of canagliflozin in relation to meal intake was unable to be assessed.

Overall, this case series did not show that canagliflozin was effective in treating PBH. This contrasts with prior studies that found an improvement in these parameters. However, these studies assessed postprandial glycemic responses within an hour after canagliflozin dosing. It is likely that the beneficial effects observed are dependent on timing of medication administration in relation to caloric intake. Larger prospective clinical trials such as the ongoing HypoBar I trial^15^ are needed to better understand and characterize the potential use of canagliflozin in patients with PBH before it can be considered as a possible treatment option.

## Disclosure

H.M.L. received funding as a principal investigator for Vogenx, Inc., and served as an advisory board member for Amylyx Pharmaceuticals. The other author has no conflicts of interest to disclose.
